# Analysis on topological alterations of functional brain networks after acute alcohol intake using resting-state functional magnetic resonance imaging and graph theory

**DOI:** 10.3389/fnhum.2022.985986

**Published:** 2022-09-26

**Authors:** Gengbiao Zhang, Hongkun Liu, Hongyi Zheng, Ni Li, Lingmei Kong, Wenbin Zheng

**Affiliations:** ^1^Department of Radiology, The Second Affiliated Hospital, Shantou University Medical College, Shantou, China; ^2^The Family Medicine Branch, Department of Radiology, The First Affiliated Hospital, Shantou University Medical College, Shantou, China

**Keywords:** alcohol, functional magenetic resonance imaging, graph theory, breath alcohol concentration, brain functional network, healthy volunteers

## Abstract

**Aims:**

Alcohol consumption could lead to a series of health problems and social issues. In the current study, we investigated the resting-state functional brain networks of healthy volunteers before and after drinking through graph-theory analysis, aiming to ascertain the effects of acute alcohol intake on topology and information processing mode of the functional brain networks.

**Materials and methods:**

Thirty-three healthy volunteers were enrolled in this experiment. Each volunteer accepted alcohol breathalyzer tests followed by resting-state magnetic resonance imaging at three time points: before drinking, 0.5 h after drinking, and 1 h after drinking. The data obtained were grouped based on scanning time into control group, 0.5-h group and 1-h group, and post-drinking data were regrouped according to breath alcohol concentration (BrAC) into relative low BrAC group (A group; 0.5-h data, *n* = 17; 1-h data, *n* = 16) and relative high BrAC group (B group; 0.5-h data, *n* = 16; 1-h data, *n* = 17). The graph-theory approach was adopted to construct whole-brain functional networks and identify the differences of network topological properties among all the groups.

**Results:**

The network topology of most groups was altered after drinking, with the B group presenting the most alterations. For global network measures, B group exhibited increased global efficiency, Synchronization, and decreased local efficiency, clustering coefficient, normalized clustering coefficient, characteristic path length, normalized characteristic path length, as compared to control group. Regarding nodal network measures, nodal clustering coefficient and nodal local efficiency of some nodes were lower in B group than control group. These changes suggested that the network integration ability and synchrony improved, while the segregation ability diminished.

**Conclusion:**

This study revealed the effects of acute alcohol intake on the topology and information processing mode of resting-state functional brain networks, providing new perceptions and insights into the effects of alcohol on the brain.

## Introduction

Alcohol is a common stimulant. Ethanol as one of the main components of alcohol is a small-molecular, lipid-soluble compound. It can enter the brain with the blood and then easily cross the blood-brain barrier and injure brain cells, resulting in damages to the brain function. According to the World Health Organization statistics (Geneva: World Health Organization, 2018), the unique flavor, euphoria-inducing and addictive properties of alcohol have turned approximately 2.3 billion people around the world into drinkers, including more than half of the population from the Americas, Europe, and Western Pacific. In the meantime, the side effects brought by harmful drinking have badly affected people’s health and social stability. Statistically, harmful drinking was responsible for approximately 3 million deaths globally (5.3% of all deaths) in 2016, of which about 900 thousand deaths were attributable to alcohol. It has been reported that alcohol-related deaths are mostly associated with the changes in cognitive, emotional, and behavioral functions following the one-time alcohol overconsumption. In addition, alcoholic intoxication is usually the root of a series of health problems [mental disorder (Puddephatt et al., 2021), cardiovascular disease (Millwood et al., 2019), etc.] and social issues [violence (NORSTRöM and Pape, 2010), sexual misconduct (Fritz et al., 2010), etc.]. Therefore, it is of great significance to study brain function alterations after acute alcohol intake.

Blood oxygen level-dependent (BOLD) resting-state functional magnetic resonance imaging (rfMRI) is often used in alcohol-brain studies, attributed to characteristics such as convenient data acquisition, non-invasive manner in detection of brain activity changes and availability of multiple brain activity parameters. However, most of the studies focused on alcoholic encephalopathy and corresponding brain alterations resulted from chronic alcoholism (Fritz et al., 2019). Recent years have witnessed an increasing number of studies relevant to acute alcoholism (Nikolaou et al., 2013; Spagnolli et al., 2013; Zheng et al., 2015), but most of them were concentrated on specific brain regions or sub-networks after alcohol intake and the relationship between imaging findings and brain function. It is a fact that performance of higher brain functions such as the cognition, memory, and emotional functions is highly dependent on the coordination of multiple brain regions and sub-networks. In this context, the existing studies still have some limitations.

Graph-theory analysis is emerging as a novel approach in studies concerning functional brain networks using imaging techniques. It enables the topological properties of functional brain networks to be analyzed from the macroscopic view of global brain network, putting it at an advantage over other analytic methods (Sporns et al., 2005; Petersen and Sporns, 2015). Currently, it has been widely adopted in the studies on the functional brain networks in either healthy or diseased persons (Bullmore and Sporns, 2009; He and Evans, 2010; Rubinov and Sporns, 2010). Additionally, applications in alcohol-brain studies have been also reported (Lithari et al., 2012; Sjoerds et al., 2017; Kimbrough et al., 2020; Smith and Kimbrough, 2020). Under this background, graph-theory analysis has its unique value in studies concerning the mechanism for alcohol action on the human brain, and related experiments may offer new perspectives and ideas for the exploration of effects brought by acute alcohol intake on brain functions.

The present study intended to construct the resting-state functional brain networks of healthy volunteers at different time points before and after drinking using rfMRI. In the meantime, graph-theory analysis was performed to analyze the topological properties of the networks. Given the individual differences brought by variations in alcohol absorption and metabolic rates (Yoshida, 1983; Baraona et al., 2001), data of different time points after drinking were sub-grouped based on the breath alcohol concentration (BrAC) into A group with relative low BrAC and B group with relative high BrAC, for more detail about the grouping, please refer to section “Statistical analysis.” In that way, the topological alterations of functional brain networks at varying conditions were investigated, which may provide information for revealing the information processing mode of functional brain network after drinking and suggest a theoretical basis for the exploration of mechanism for alcohol action on the brain and for the prevention of secondary injuries caused by drinking.

## Materials and methods

### Participants

Thirty-three student volunteers (17 males and 16 females), aged 23–31 (mean: 25.42 ± 1.80 years), right-handed, were recruited from Shantou University Medical College. All of them had no previous history of major diseases or alcohol abuse, family history of psychiatric disorders or contraindications to MRI. The volunteers were required to avoid anesthetic and psychotropic drugs for 1 month before the experiment, to abstain from coffee, strong tea, and all alcoholic food or beverages for 24 h before the experiment, and to refrain from eating for 6 h before the experiment. Besides, alcohol breathalyzer test was performed to ensure volunteers had not ingested alcohol prior to the experiment.

The experimental protocol was approved by the Ethics Committee of Shantou University Medical College. All the volunteers were fully informed of the amount of alcohol consumption and precautions for MRI and signed the informed consent form.

### Procedure

The volunteers were instructed to finish drinking 0.65 g/kg alcohol (0.65 g alcohol/1 kg body weight) within 6–10 min. Each participant had a tailored alcohol dose based on body weight: alcohol consumption (milliliters) = the amount of alcohol intake (grams)/alcohol concentration (percentage) × 0.8 (ethanol attenuation). The Chinese Baijiu “Qinghua Fenjiu 20” (53% VOL) produced in Shanxi Province, China, together with some food (biscuits, peanuts, etc.), was offered. Each volunteer accepted alcohol breathalyzer tests followed by MRI examinations at three time points: before drinking, 0.5 h after drinking, and 1 h after drinking. BrAC was detected by Alco-Sensor III breathalyzer (ALCPRO, Knoxville, Tennessee) (Borges et al., 2004) and the blood alcohol concentration was estimated accordingly. Thin-slice T1-weighted image and BOLD image data were collected before drinking, while only BOLD image data were collected 0.5 and 1 h after drinking. The volunteers were required to report their subjective feelings such as dizziness, headache, and euphoria and the presence of blushing at each time point.

### Magnetic resonance imaging acquisition

MRI was performed on all subjects using a 3.0-T GE MRI system (Signa; General Electric Medical System, USA) with an eight-channel head coil (GE Medical Systems). All subjects underwent Ax 3D-BRAVO (three-dimensional brain volume imaging) T1-weighted imaging scan base on gradient echo sequence to determine whether there were anatomical abnormalities of the brain before the rfMRI scans, the imaging parameters were set as follows: slice thickness = 1.2 mm, slice gap = 0 mm, TR/TE = 7.8/3 ms, FOV = 24 cm × 24 cm, flip angle = 15°, matrix = 256 × 256, 248 slices.

BOLD rfMRI data were acquired using gradient-echo echo-planar imaging sequences (slice thickness = 5 mm, slice gap = 0 mm, TR/TE = 2,000/30 ms, FOV = 24 cm × 24 cm, flip angle = 90°, matrix = 64 × 64, 25 slices) to measure the individual fMRI data. The duration of the resting-state BOLD scan was 7 min, and it produced 210 frames (5,250 DICOM images). The subjects were told to close their eyes and not to fall asleep during the scanning.

### Image preprocessing

Statistical Parametric Mapping (SPM) 12^1^ based GRETNA toolbox^2^ was adopted to pre-process rfMRI data. Firstly, DICOM images were converted to NIFTI format. In order to allow for MR signal to stabilize, data of the first 10 time points were removed and the slice time and head motion (in three directions, translation < 3 mm, rotation < 3°) were corrected. Then, the fMRI images were spatially normalized to the Montreal Neurological Institute (MNI) EPI template, and resampled to 3 mm cubic voxels. The Friston-24 head motion parameters (motion-related effects in fMRI time series) and other confounds (global signal, white matter, and cerebrospinal fluid) were regressed for each voxel. Finally, linear drift was removed and the signals were bandpass filtered (0.01–0.1 Hz).

### Network construction

**FIGURE 1:**
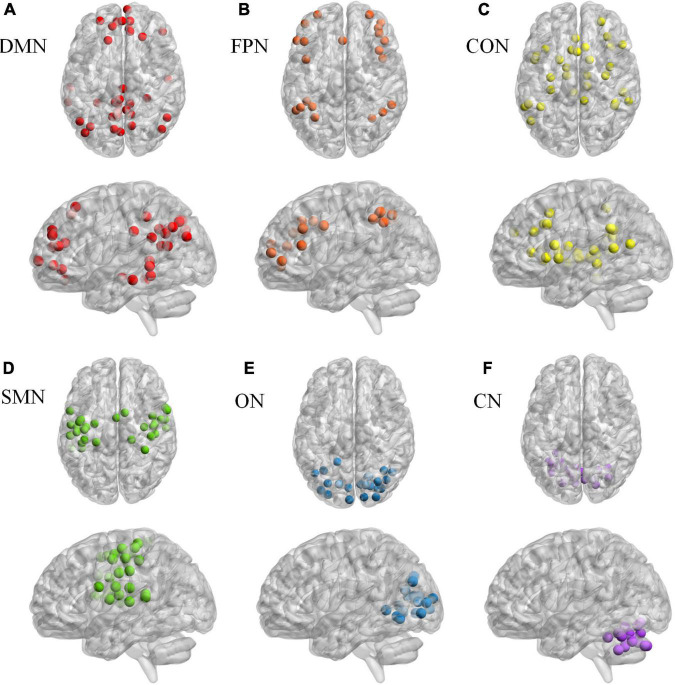
The Dosenbach 160 atlas includes 160 brain regions, which are classified into 6 sub-networks including Default Mode Network **(A)**, Fronto-parietal Network **(B)**, Cingulo-opercular Network **(C)**. Sensorimotor Network **(D)**, Occipital Network **(E)**, and Cerebellum Network **(F)**.

**FIGURE 2:**
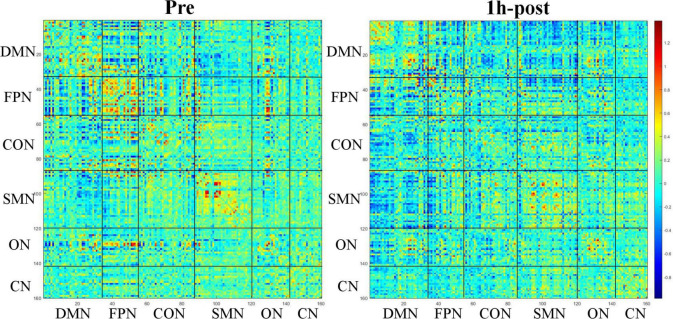
Functional connectivity matrix obtained from a 31-year-old male participant before and 1 h after drinking. The color of the blocks in the matrix represents the connection strength of the nodes where the horizontal and vertical axes intersect.

Based on Dosenbach 160 atlas (Dosenbach et al., 2010), the brain was parcellated into 160 regions of interest (ROIs) through meta-analysis, which were further assigned to 6 functional sub-networks, including Default Mode Network (DMN), Fronto-parietal Network (FPN), Cingulo-opercular Network (CON), Sensorimotor Network (SMN), Occipital Network (ON), and Cerebellum Network (CN) (Figure 1 for more information about the 160 ROIs, please refer to Supplementary File 1). Then, we extracted the mean time series for each node and computed the Pearson correlation coefficient between each node pair to evaluate the inter-regional resting-state functional connectivity of each participant. Finally, correlation coefficients were converted to a normal distribution using Fisher’s r-to-z transform (Figure 2).

### Network analysis

Sparsity is the ratio of the number of existing edges to the maximum possible number of edges in a network. Setting of sparsity thresholds can guarantee all the constructed networks have the same number of edges, enabling us to detect the inter-group differences. Based on previous studies (Yang et al., 2021), the sparsity threshold range was identified as 0.1–0.34 at intervals of 0.01 in this experiment.

Global and nodal measures at different sparsity thresholds were calculated. The global measures included five small-world network parameters (clustering coefficient C_p_, characteristic path length L_p_, normalized clustering coefficient γ, normalized characteristic path length λ, and small-worldness σ), two efficient network parameters (local efficiency E_loc_ and global efficiency E_glob_), Assortativity and Synchronization. C_p_, γ, and E_loc_ are measures of network segregation, L_p_, λ, and E_glob_ are measures of network integration, while σ is measures of the efficiency of information transfers (Rubinov and Sporns, 2010). Assortativity represents for network resilience (Newman, 2002), and Synchronization reveals the possibility of all the nodes with identical waveform (Wang et al., 2015). Nodal measure included nodal degree, nodal betweenness, nodal clustering coefficient, nodal efficiency (nodal local efficiency and nodal global efficiency) and nodal characteristic path length. Table 1 provides mathematical definitions of network metrics.

**TABLE 1:** Mathematical definitions of network metrics.

Parameters	Formula	Basic concepts and notation
Global efficiency, E_glob_ (Latora and Marchiori, 2001)	$E_{\text{glob}}=\frac{1}{\text{n}}\,\sum_{\text{i}\in N}E_{i}=\frac{1}{\text{n}}\,\sum_{i\in N}\frac{\sum_{j\in N,j\neq i}d_{i\,j}^{-1}}{n-1}$	1. N is the set of all nodes in the network, and n is the number of nodes. 2. L is the set of all links in the network, and l is number of links. 3. (i, j) is a link between nodes i and j, (i, j∈ N). 4. a_ij_ is the connection status between i and j: a_ij_ = 1 when link (i, j) exists (when i and j are neighbors); a_ij_ = 0 otherwise (a_ii_ = 0 for all i). 5. The number of links is l = ∑_i_,_j∈__N_ a_ij_ (to avoid ambiguity with directed links, each undirected link is counted twice, as a_ij_ and as a_ji_). 6. E_i_ is the efficiency of node i; E_loc_,_i_ is the local efficiency of node i, and d_jh_ (N_i_) is the length of the shortest path between j and h, that contains only neighbors of i; C_i_ is the clustering coefficient of node i (C_i_ = 0 for k_i_ < 2). 7. C and C_rand_ are the clustering coefficients, and L and L_rand_ are the characteristic path lengths of the respective tested network and a random network. Small-world networks often have S> 1. 8. G_2_ and G_N_ are the second smallest and the largest eigenvalue of the coupling matrix G which is defined as: $G_{\text{ij}}=\delta_{\text{ij}}\,\sum_{\text{i}=1}^{\text{N}}{\text{a}}_{\mathrm{ij}-}\,{\text{a}}_{\text{ij}}$ 9. σ_mn_ is the total number of shortest paths (paths with the shortest path length) from node m to node n, and σ_mn_(i) is the number of shortest paths from node m to node n that pass through the node i.
Local efficiency, E_loc_ (Latora and Marchiori, 2001)	$\begin{array}{c} E_{\text{loc}}=\frac{1}{\text{n}}\,\sum_{\text{i}\in N}E_{l\,o\,c,i}\\ =\frac{1}{\text{n}}\,\sum_{i\in N}\frac{\sum_{j,h\in N,j\neq i}a_{i\,j}\,a_{i\,h}\,{\left[d_{j\,h}\,\left(N\,i\right)\right]}^{-1}}{k_{i}\,\left(k_{i}-1\right)} \end{array}$	
Clustering coefficient, C_p_ (Watts and Strogatz, 1998)	$C_{\text{p}}=\frac{1}{n}\,\sum_{i\in N}C_{\text{i}}=\frac{1}{n}\,\sum_{i\in N}\frac{2\,t_{i}}{k_{i}\,\left(k_{i}-1\right)}$	
Characteristic path length, L_p_ (Watts and Strogatz, 1998)	$L_{\text{p}}=\frac{1}{\text{n}}\,\sum_{\text{i}\in N}\frac{\sum_{j\in N,j\neq i}d_{i\,j}}{n-1}$	
Normalized clustering coefficient, γ	_γ=C_p/C_p–rand_	
Normalized characteristic path length, λ	_λ=L_p/L_p–rand_	
Small-worldness, σ (Humphries and Gurney, 2008)	σ = γ/λ	
Assortativity, r (Newman, 2002)	$r=\frac{l^{-1}\,\sum_{\left(i,j\right)\in L}k_{i}\,k_{j}-{\left[l^{-1}\,\sum_{\left(i,j\right)\in L}\frac{1}{2}\,\left(k_{i}+k_{j}\right)\right]}^{2}}{l^{-1}\,\sum_{\left(i,j\right)\in L}\frac{1}{2}\,\left(k_{i}^{2}+k_{j}^{2}\right)-{\left[l^{-1}\,\sum_{\left(i,j\right)\in L}\frac{1}{2}\,\left(k_{i}+k_{j}\right)\right]}^{2}}$	
Synchronization, S (Barahona and Pecora, 2002; Motter et al., 2004)	*S* = *G*_2_/*G*_*N*_	
Nodal betweenness, b_i_ (Freeman, 1977)	$b_{i}=\sum_{m\neq i\neq n\in G}\frac{\sigma_{m\,n}\,\left(i\right)}{\sigma_{m\,n}}$	
Nodal degree, *k*	k_i_ = ∑_*j*∈*N*_*a*_*ij*_	

Area under curve (AUC) for each network measure within the sparsity thresholds (0.1–0.34 at intervals of 0.01) was calculated to provide a summarized scalar (independent from single threshold selection) with favorable sensitivity for the topological characterization of brain networks.

### Statistical analysis

Post-drinking data, including data after 0.5 and 1 h of drinking, were sub-grouped by BrAC into A group and B group. Data of each volunteer with relative higher BrAC at two time points after drinking were included in B group, and other data were included in A group. Which means that B group will include the data of 33 volunteers at different time points when the BrAC is relative higher at two time points, while A group will include the data when the BrAC is relative lower. Pre-drinking data served as control. This grouping aim to ensure that the data of B group are the data captured when the BrAC of each volunteer is the highest under the current experimental conditions, the results of B group can represent the state of all volunteers when their BrAC is high, and excluding the influence of individual differences on the experiment to the greatest extent possible.

Demographic data were compared using Statistical Package of the Social Sciences (SPSS) 26.0 (IBM, Armonk, New York, USA). Repeated-measured analysis of variance (ANOVA) was used to analyze the inter-group differences regarding global network parameters if the data were normally distributed, otherwise Friedman test was applied. Bonferroni correction was utilized for multiple comparisons. Nodal network parameters were compared via paired-sample *T*-test in GRETNA toolbox, and multiple comparisons were corrected with false discovery rate (FDR). As pairwise comparisons were performed between nodal network parameters in this experiment, the P threshold generated by GRETNA divided by 3 was taken as the corrected threshold to identify nodes with statistical differences. To localize those neural circuits showing significant changes in functional connectivity strength, the Network-Based Statistic (NBS) method^3^ was used. A non-parametric permutation test (threshold = 2.6, *p* < 0.05, 10,000 permutations) was adopted to identify significant inter-group differences in functional connectivity strength.

## Results

### Clinical manifestations and grouping

After acute alcohol intake, some people presented changes in mood, behavior and skin of varying degrees, including dizziness (17/33), headache (2/33), excitement (6/33), unsteady gait (5/33), depression (1/33), nausea (3/33), and blush (16/33). According to BrAC, the post-drinking data were sub-divided into B group (0.5-h data, *n* = 16; 1-h data, *n* = 17) and A group (0.5-h data, *n* = 17; 1-h data, *n* = 6). Some volunteers in B group reported more obvious symptoms than A group 0.5 and 1 h after drinking, including severer dizziness, headache and nausea and more evident mood swings. All the volunteers showed slight to moderate intoxication.

### Global measures

**FIGURE 3:**
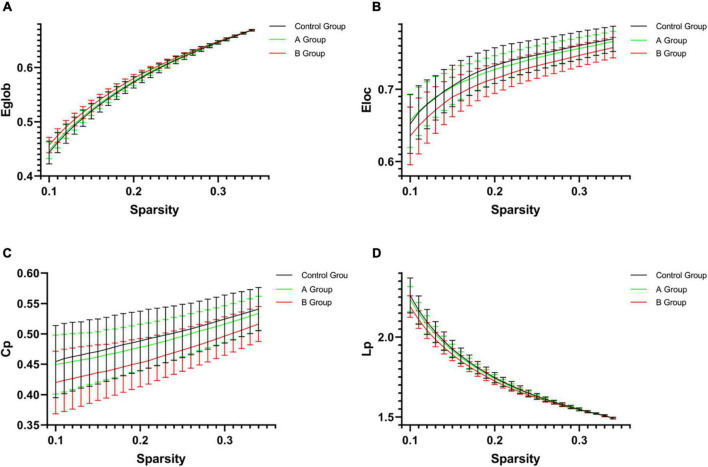
The network parameters [**(A)** Eglob; **(B)** Eloc; **(C)** Cp; and **(D)** Lp] of control group, A group and B group within a given threshold range. Error bars represent standard errors. As compared with control group, B group exhibited increased E_glob_ (*p* = 0.004) but decreased E_loc_ (*p* = 0.001), C_p_ (*p* = 0.001), and L_p_ (*p* = 0.003). When compared with A group, B group presented increased E_glob_ (*p* = 0.001) but decreased E_loc_ (*p* < 0.001), C_p_ (*p* < 0.001), and L_p_ (*p* = 0.001). E_glob_, global efficiency; E_loc_, local efficiency; Cp, clustering coefficient; Lp, characteristic path length.

As compared with control group, 0.5 h group presented decreased E_loc_ (*p* = 0.036), C_p_ (*p* = 0.026), L_p_ (*p* = 0.019), λ (*p* = 0.031), and increased E_glob_ (*p* = 0.050), 1 h group showed decreased E_loc_ (*p* = 0.019) and L_p_ (*p* = 0.036), while B group exhibited increased E_glob_ (*p* = 0.004) and Synchronization (*p* = 0.007) but decreased E_loc_ (*p* = 0.001), C_p_ (*p* = 0.001), L_p_ (*p* = 0.003), γ (*p* = 0.012), λ (*p* = 0.003), and σ (*p* = 0.027) (Table 2 and Figure 3). When compared with A group, B group presented increased E_glob_ (*p* = 0.001) and Synchronization (*p* = 0.014) but decreased E_loc_ (*p* < 0.001), C_p_ (*p* < 0.001), L_p_ (*p* = 0.001), γ (*p* < 0.001), λ (*p* = 0.001), and σ (*p* = 0.001) (Table 2 and Figure 3). No statistical differences were found in other comparisons.

**TABLE 2:** Global network parameters in each group.

Parameters	Control group	0.5 h group	1 h group	A group	B group
Eglob	0.1391 ± 0.0022	0.1400 ± 0.0017*	0.1400 ± 0.0016	0.1396 ± 0.0014	0.1405 ± 0.0017**^§§^
Eloc	0.1755 ± 0.0058	0.1729 ± 0.0057*	0.1734 ± 0.0045*	0.1745 ± 0.0045	0.1718 ± 0.0053**^§§§^
Cp	0.1190 ± 0.0112	0.1138 ± 0.0103*	0.1143 ± 0.0084	0.1165 ± 0.0082	0.1115 ± 0.0099**^§§§^
Lp	0.4195 ± 0.0078	0.4161 ± 0.0058*	0.4160 ± 0.0054*	0.4175 ± 0.0049	0.4147 ± 0.0059**^§§^
γ	0.5073 ± 0.0396	0.4956 ± 0.0407	0.5013 ± 0.0307	0.5083 ± 0.0349	0.4887 ± 0.0346*^§§§^
λ	0.2513 ± 0.0040	0.2496 ± 0.0030*	0.2497 ± 0.0028	0.2503 ± 0.0026	0.2489 ± 0.0030**^§§^
σ	0.4808 ± 0.0324	0.4731 ± 0.0340	0.4785 ± 0.0255	0.4836 ± 0.0294	0.4680 ± 0.0289*^§§^
Assortativity	0.0582 ± 0.0226	0.0571 ± 0.0210	0.0543 ± 0.0202	0.0586 ± 0.0207	0.0528 ± 0.0201
Synchronization	0.0304 ± 0.0093	0.0322 ± 0.0109	0.0336 ± 0.0085	0.0301 ± 0.0089	0.0357 ± 0.0099**^§^

AUC of the parameters of nine global topologies in five groups. Values represent mean ± SD. Comparison between groups, AUC values with significant effects after correction for multiple comparisons (*p* < 0.05, Bonferroni corrected) are in bold (comparison before and after drinking, *p < 0.05; **p < 0.01; comparison between A Group and B Group, ^§^*p* < 0.05; ^§$^*p* < 0.01; ^§§§^*p* < 0.001). Cp, clustering coefficient; γ, normalized clustering coefficient; λ, normalized characteristic path length; Lp, characteristic path length; σ, small-worldness; Eglob, global efficiency; Eloc, local efficiency.

### Nodal measures

**FIGURE 4:**
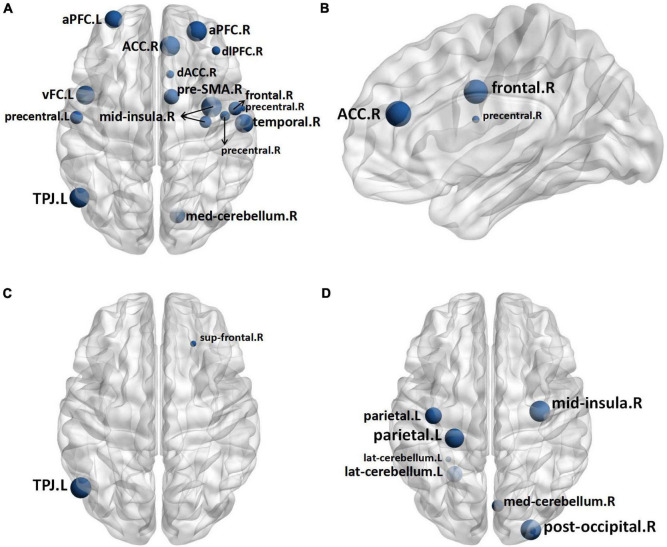
In comparison to the Control Group, the nodes in the B Group with decreased nodal clustering coefficient **(A)** and nodal local efficiency **(B)**, and the nodes in the 1 h Group with decreased nodal clustering coefficient **(C)**. In comparison to Group A, the nodes in the B Group have a lower nodal clustering coefficient **(D)**. The size of the nodes represents the level of significance. Sup frontal, superior frontal gyrus; TPJ, temporo-parietal junction area; ACC, anterior cingulate cortex; aPFC, anterior prefrontal cortex; dACC, dorsal anterior cingulate cortex; vFC, ventral frontal cortex; mid insula, mid-insula; pre-SMA, presupplementary motor area; precentral, precentral gyrus; temporal, temporal lobe; med cerebellum, median cerebellum; parietal, parietal gyrus; post occipital, posterior occipital gyrus; lat cerebellum, lateral cerebellum.

Two nodes were identified to have statistical differences between 1 h group and control group. As compared with control group, 1 h group exhibited lower nodal clustering coefficient in right superior frontal gyrus (sup frontal.R) and left temporo-parietal junction area (TPJ.L) (Figure 4C). The statistical differences between the B group and control group were found in sixteen nodes, B group exhibited lower nodal clustering coefficient in right anterior cingulate cortex (ACC.R), bilateral anterior prefrontal cortex (aPFC.B), dorsolateral prefrontal cortex (dlPFC.R), dorsal anterior cingulate cortex (dACC.R), left ventral frontal cortex (vFC.L), right mid-insula (mid insula.R), TPJ.L, right presupplementary motor area (pre-SMA.R), right frontal cortex (frontal.R), bilateral precentral gyrus (precentral.B), right temporal lobe (temporal.R) and right median cerebellum (med cerebellum.R), and showed decreased nodal local efficiency in dACC.R, frontal.R, and precentral gyrus.R (Figures 4A,B). Seven nodes were identified to have statistical differences between A group and B group, B group exhibited lower nodal clustering coefficient in mid-insula.R, left parietal gyrus (parietal.L), right posterior occipital gyrus (post occipital.R), left lateral cerebellum (lat cerebellum.L), and med cerebellum.R (Figure 4D). No statistical differences were identified in other comparisons (for more information about the nodal measures, please refer to Supplementary Table 1).

### Functional connectivity strength

**FIGURE 5:**
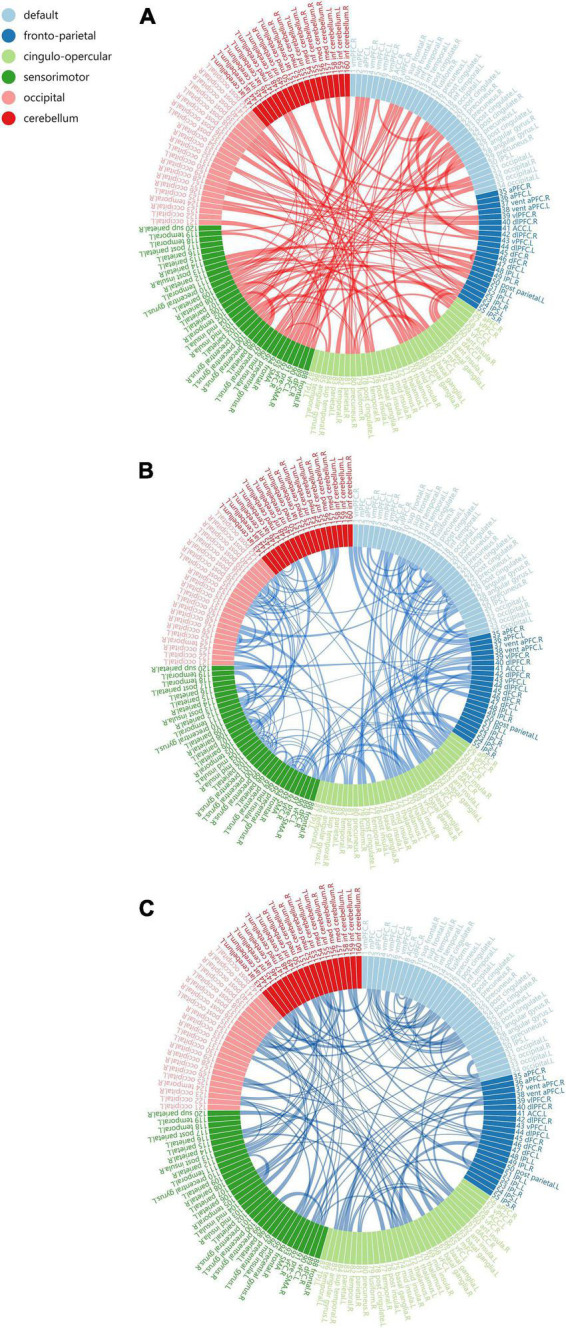
Compared with Control Group (*p* < 0.05, corrected). The neural circuits with altered functional connectivity strength in B Group **(A,B)** and A Group **(C)**. Edge colors represent increased (red) and decreased (blue) functional conneetivity strength in the volunteers. Edge sizes represent the level of significance (for more information about the functional connectivity changes, please refer to Supplementary File 3).

There were neural circuits differing statistically from control group in A group and B group. A group’s circuit included 110 edges and 93 nodes, all of which appeared to be weakened (Figure 5C). We identified two neural circuits in the B group, one had 103 nodes and 111 edges, and all of its edges displayed increasing connection strength (Figure 5A). The other circuit had 118 nodes and 149 edges, and all of its edges displayed a weakening of connection strength (Figure 5B). In addition, group B showed two statistically different neural circuits compared to group A. One of the circuit includes 94 nodes and 106 edges, all of which show increased connection strength, and the other circuit includes 112 nodes and 151 edges, all of which show weaker connections. Compared with the control group and A group, the edges in the neural circuits with increased connection strength in B group were more represented as inter-network connections. In the neural circuits with weakened connections, the circuits involving B group showed a larger proportion of weakened intra-network connections (Table 3). No edges with statistical differences were observed in other between-group comparisons.(for more information about the functional connectivity changes, please refer to Supplementary Figure 1 and Supplementary File 3).

**TABLE 3:** Number of different types of edges in each neural circuits.

Neural circuits	Intra-network connection	Inter-network connection	Total
B group > Control group	7 (6.3%)	104 (93.7%)	111 (100.0%)
B group < Control group	60 (40.0%)	89 (60.0%)	149 (100.0%)
B group > A group	11 (10.4%)	95 (89.6%)	106 (100.0%)
B group < A group	74 (49.0%)	77 (51.0%)	151 (100.0%)
A group < Control group	29 (26.4%)	81 (73.6%)	110 (100.0%)

The parentheses show the proportions of different types of edges in the neural circuits in which they are located (for more information about the functional connectivity changes, please refer to Supplementary File 3).

## Discussion

In the current study, we investigated the resting-state functional brain networks of healthy volunteers before and after drinking through graph-theory analysis, aiming to ascertain the effects of acute alcohol intake on functional brain network topology and revealing the information processing mode of functional brain network after drinking.

### Global measure alterations

Functional integration and segregation, two major organizational principles of human brain (Tononi et al., 1998), allow the brain to extract and process information rapidly. Functional integration refers to the ability of combining the specialized information from various brain regions rapidly, while functional segregation is the ability to process information in a specialized way within connected brain regions (Rubinov and Sporns, 2010). In terms of global network measures, B group showed significantly increased E_glob_ and evidently decreased E_loc_, L_p_, C_p_, λ, and γ, as compared with the control group and A group. These changes demonstrated that acute alcohol intake altered the information processing mode of resting-state functional brain networks, leading to enhanced integration ability and decreased segregation ability. As compared with control group, E_glob_ increased and E_loc_, C_p_, L_p_, λ decreased in 0.5 h group, E_loc_ and L_p_ reduced in 1 h group, which was also a manifestation of enhanced integration ability and weakened segregation ability of the functional network. Combining the findings, we found that the effects of alcohol on functional network topological properties were more prominent when blood alcohol concentration was higher. Additionally, B group presented higher Synchronization than control group and A group, suggesting a convergence in the fluctuations between brain regions.

Considering that the functions of drunken volunteers including cognition, memory, movement, and sensation diminished in varying degrees, we speculated that the weakened network segregation ability might be the leading cause of the clinical manifestations of the volunteers and the improved integration ability and synchrony worked as the compensated alterations for maintaining the normal operation of functional networks. Previous studies (Mihic and Harris, 1996; Fingelkurts et al., 2004; Lithari et al., 2012) revealed that the alterations of information processing mode of functional networks after drinking might be correlated with alcohol-sensitive gamma aminobutyric acid (GABA). The interaction of alcohol and GABA_A_ receptors on cell membranes can inhibit the neuronal activity, leading to weakening of the network segregation ability while strengthening of inter-regional functional connectivity, which thereby enhances the integration ability of functional networks and synchrony between brain regions. Compelling evidence is also provided by studies of Lithari et al. (2012) and Cao et al. (2014) The current study also noted that the B group exhibited lower σ than control group and A group, indicating that the information transfer efficiency of functional brain networks declined after drinking, making the relative brain networks potentially more susceptible to dysfunction, which is consistent with the clinical manifestations of volunteers after drinking.

### Nodal measure alterations

In terms of nodal network measures, 16 nodes presenting significant differences in topological properties were identified in B group and control group. Some B regions presented decreased nodal local efficiency (*n* = 3/16) and all regions presented decreased nodal clustering coefficient, which were present concurrently in the dACC.R, frontal.R, and precentral gyrus.R. All the nodes were characterized by diminished nodal network segregation ability, consistent with the global measures.

Most of these differential nodes belonged to SMN (43.8%) and CON (31.3%), and a small number of the nodes belonged to FPN (12.5%), DMN (6.3%), and CN (6.3%). SMN mainly manages somatosensory and motor abilities, CON, FPN, and DMN are believed to jointly control working memory, attention, decision-making, and other advanced cognitive abilities through interactions (Dosenbach et al., 2007; Sonuga-Barke and Castellanos, 2007; Anticevic et al., 2012), while CN is primarily associated with functions such as sensory-motor coordination, emotion, and cognition (De Benedictis et al., 2022). The alterations in topology of brain regions and function of sub-networks are highly consistent with the impaired functions induced by drinking, which further supports our estimation that “weakened network segregation ability is the leading cause of the clinical manifestations of the volunteers.” Additionally, we also noted that alcohol might mainly act on dACC.R, frontal.R, and precentral gyrus.R, three brain regions with the most significant topological alterations. Studies (Dosenbach et al., 2007) revealed that CON and FPN constituted a system responsible for the top-down control over behaviors. In this context, the post-drinking decline in self-control might be partly due to the simultaneous decrease in segregation ability of CON and FPN.

It is noteworthy that there were no nodes with advanced network integration ability in all the groups, which might be on account of the stringent thresholds selected in the analysis of nodal network parameters.

### Functional connectivity differences

Edge analysis suggested that acute alcohol exposure can significantly alter the connectivity status of functional brain networks. Compared with the control group, the neural circuit of A group showed weakened connection strength, while B group showed both increased and weakened connection strength. By classifying the changed edges into two types: intra-network connections where the connection nodes belong to the same sub-network and inter-network connections where the connection nodes belong to different sub-networks, we find that neural circuits with altered connection states are characteristic in the distribution of connection types. First, compared with the control group and A group, the edges in the neural circuits with increased connection strength in B group were more represented as inter-network connections, suggesting that the information exchange between different sub-networks increased, which was consistent with the significantly higher network integration ability of B group. However, considering that the functional network also has weakened inter-network connections after drinking, the improvement of integration ability is more likely to be the comprehensive result of the mutual influence of different types of inter-network connections after network rearrangement. This argument is also supported by the fact that in the B group, there are more edges with enhanced inter-network connections than with weakened inter-network connections. Second, in the neural circuits with weakened connections, both groups A and B showed different numbers of weakened intra-network connections, suggesting that the internal information exchange of the sub-networks was reduced. However, the circuits involving B group showed a larger proportion of weakened intra-network connections, which is consistent with the weaker network segregation ability of B group. The relative scarcity of edges exhibiting enhanced connectivity within the sub-network in circuits involving B group also supports this hypothesis.

In conclusion, alcohol may affect the integration and segregation abilities of functional brain networks by modulating different types of network connections. The improved integration ability was attributed to the increased connectivity between different sub-networks, while the weakened segregation ability was the result of the weakened intra-network connectivity. It suggests that the functional connection types of sub-networks is correlated with topological properties, and there may be a mutual influence relationship. Combining the two may better characterize the information processing mode of functional brain network.

### Information processing mode alterations

By combining the results of each section, we tried to deduce the mechanism of alcohol’s effect on the information processing mode of functional brain network, and explain the relationship between the change of information processing mode and clinical manifestations of volunteers. Alcohol inhibits neuronal activity by interacting with GABA_A_ receptors on cell membranes and leads to strengthening of inter-regional functional connectivity. These changes are manifested in the enhanced inter-network connections and weakened intra-network connections in the network connection state, and in the topology of functional brain network showed that the network segregation ability and information transfer efficiency diminished, while the integration ability and synchrony improved. Combined with the results that Cp, Lp, and σ all decreased, we found that the topology of functional brain network changed to random network after drinking (Bassett and Bullmore, 2017). This means that the network is more unstable, and at the same processing efficiency, the functional brain network after drinking requires more cost to keep functioning. Drinking can change or even damage the information processing mode of functional brain network, and the deterioration of information processing mode is manifested as the impairment of volunteers’ function (cognition, movement, sensation, self-control, etc.), which is also the main reason for secondary injury after drinking. Previous studies (Shine and Poldrack, 2018) have shown that the imbalance of separation ability and integration ability of brain network can affect individual task performance, which further supports our theory. Our findings may provide a theoretical basis for the development of drugs or therapies to curb inappropriate behavior (violence, drunk driving, sexual misconduct, etc.) after drinking.

### Limitations

This study has several limitations. More subjects of different ages and longer observation periods are needed to further investigate the potential effects of age, sex, time, and blood alcohol concentration. And we believe that increasing the sample size of the study will enrich the fMRI data to achieve better results. In addition, the current study chose a strict statistical strategy in this experiment. Rigorous multiple corrections enhanced the reliability of our results, but also resulted in fewer statistically different results. We will make further analysis based on this experiment with more abundant data and more advanced technology.

## Conclusion

The current study found that acute alcohol intake could markedly alter or even damage the information processing mode of functional brain networks, mainly manifested as enhanced network integration ability and declined segregation ability, which might be the leading cause of the clinical manifestations of the volunteers. Furthermore, alcohol was also capable of advancing the synchrony of resting-state functional networks. In conclusion, this study revealed the effects of acute alcohol intake on resting-state functional network topology and information processing mode, providing new perceptions and insights into the effects of alcohol on the brain.

## Data availability statement

The raw data supporting the conclusions of this article will be made available by the authors, without undue reservation.

## Ethics statement

The studies involving human participants were reviewed and approved by the Ethics Committee of Shantou University Medical College. The patients/participants provided their written informed consent to participate in this study.

## Author contributions

WZ: conceptualization. HL, GZ, and NL: data acquisition and data curation. GZ and HL: data analysis and methodology. GZ: statistical analysis and writing—original draft. HZ, LK, and WZ: manuscript editing and manuscript review. All authors contributed to the article and approved the submitted version.

## Footnotes

1
http://www.fil.ion.ucl.ac.uk/spm/software/spm12/

2
http://www.nitrc.org/projects/gretna/

3
https://www.nitrc.org/projects/nbs

## References

[B1] Anticevic A. Cole M. W. Murray J. D. Corlett P. R. Wang X. J. Krystal J. H. (2012). The role of default network deactivation in cognition and disease. *Trends Cogn. Sci.* 16 584–592. 10.1016/j.tics.2012.10.008 23142417 PMC3501603

[B2] Barahona M. Pecora L. M. (2002). Synchronization in small-world systems. *Phys. Rev. Lett.* 89:054101. 10.1103/PhysRevLett.89.054101 12144443

[B3] Baraona E. Abittan C. S. Dohmen K. Moretti M. Pozzato G. Chayes Z. W. (2001). Gender differences in pharmacokinetics of alcohol. *Alcohol Clin. Exp. Res.* 25 502–507. 11329488

[B4] Bassett D. S. Bullmore E. T. (2017). Small-World Brain Networks Revisited. *Neuroscientist* 23 499–516. 10.1177/1073858416667720 27655008 PMC5603984

[B5] Borges G. Cherpitel C. Mittleman M. (2004). Risk of injury after alcohol consumption: A case-crossover study in the emergency department. *Soc. Sci. Med.* 58 1191–1200. 10.1016/s0277-9536(03)00290-9 14723913

[B6] Bullmore E. Sporns O. (2009). Complex brain networks: Graph theoretical analysis of structural and functional systems. *Nat. Rev. Neurosci.* 10 186–198. 10.1038/nrn2575 19190637

[B7] Cao R. Wu Z. Li H. Xiang J. Chen J. (2014). Disturbed connectivity of EEG functional networks in alcoholism: A graph-theoretic analysis. *Biomed. Mater Eng.* 24 2927–2936. 10.3233/bme-141112 25226999

[B8] De Benedictis A. Rossi-Espagnet M. C. De Palma L. Carai A. Marras C. E. (2022). Networking of the Human Cerebellum: From Anatomo-Functional Development to Neurosurgical Implications. *Front. Neurol.* 13:806298. 10.3389/fneur.2022.806298 35185765 PMC8854219

[B9] Dosenbach N. U. Fair D. A. Miezin F. M. Cohen A. L. Wenger K. K. Dosenbach R. A. (2007). Distinct brain networks for adaptive and stable task control in humans. *Proc. Natl. Acad. Sci. U.S.A.* 104 11073–11078. 10.1073/pnas.0704320104 17576922 PMC1904171

[B10] Dosenbach N. U. Nardos B. Cohen A. L. Fair D. A. Power J. D. Church J. A. (2010). Prediction of individual brain maturity using fMRI. *Science* 329 1358–1361. 10.1126/science.1194144 20829489 PMC3135376

[B11] Fingelkurts A. A. Fingelkurts A. A. Kivisaari R. Pekkonen E. Ilmoniemi R. J. KäHKöNEN S. (2004). Enhancement of GABA-related signalling is associated with increase of functional connectivity in human cortex. *Hum. Brain Mapp.* 22 27–39. 10.1002/hbm.20014 15083524 PMC6872077

[B12] Freeman L. C. J. S. (1977). A Set of Measures of Centrality Based on Betweenness. *Sociometry.* 40 35–41.

[B13] Fritz K. Morojele N. Kalichman S. (2010). Alcohol: The forgotten drug in HIV/AIDS. *Lancet* 376 398–400. 10.1016/s0140-6736(10)60884-7 20650516 PMC3015091

[B14] Fritz M. Klawonn A. M. Zahr N. M. (2019). Neuroimaging in alcohol use disorder: From mouse to man. *J. Neurosci. Res.* 100 1140–1158. 10.1002/jnr.24423 31006907 PMC6810809

[B15] Geneva: World Health Organization (2018). *Global status report on alcohol and health 2018: executive summary. (WHO/MSD/MSB/18.2). Licence: CC BY-NC-SA 3.0 IGO.* Geneva: World Health Organization

[B16] He Y. Evans A. (2010). Graph theoretical modeling of brain connectivity. *Curr. Opin. Neurol.* 23 341–350. 10.1097/WCO.0b013e32833aa567 20581686

[B17] Humphries M. D. Gurney K. (2008). Network ‘small-world-ness’: A quantitative method for determining canonical network equivalence. *PLoS One* 3:e0002051. 10.1371/journal.pone.0002051 18446219 PMC2323569

[B18] Kimbrough A. Lurie D. J. Collazo A. Kreifeldt M. Sidhu H. Macedo G. C. (2020). Brain-wide functional architecture remodeling by alcohol dependence and abstinence. *Proc. Natl. Acad. Sci. U.S.A.* 117 2149–2159. 10.1073/pnas.1909915117 31937658 PMC6994986

[B19] Latora V. Marchiori M. (2001). Efficient behavior of small-world networks. *Phys. Rev. Lett.* 87:198701. 10.1103/PhysRevLett.87.198701 11690461

[B20] Lithari C. Klados M. A. Pappas C. Albani M. Kapoukranidou D. Kovatsi L. (2012). Alcohol affects the brain’s resting-state network in social drinkers. *PLoS One* 7:e48641. 10.1371/journal.pone.0048641 23119078 PMC3485329

[B21] Mihic S. J. Harris R. (1996). “Alcohol actions at the GABAA receptor/chloride channel complex,” in *Pharmacological Effects of Ethanol on the Nervous System*, eds Deitrich R. A. Erwin V. G. (Boca Raton, FL: CRC Press), 51–72.

[B22] Millwood I. Y. Walters R. G. Mei X. W. Guo Y. Yang L. Bian Z. (2019). Conventional and genetic evidence on alcohol and vascular disease aetiology: A prospective study of 500 000 men and women in China. *Lancet* 393 1831–1842. 10.1016/s0140-6736(18)31772-0 30955975 PMC6497989

[B23] Motter A. E. Zhou C. Kurths J. J. V. (2004). Enhancing complex-network synchronization. *Europhys. Lett.* 22 1820–1825.

[B24] Newman M. E. (2002). Assortative mixing in networks. *Phys. Rev. Lett.* 89:208701. 10.1103/PhysRevLett.89.208701 12443515

[B25] Nikolaou K. Field M. Critchley H. Duka T. (2013). Acute alcohol effects on attentional bias are mediated by subcortical areas associated with arousal and salience attribution. *Neuropsychopharmacology* 38 1365–1373. 10.1038/npp.2013.34 23361162 PMC3656379

[B26] NORSTRöM T. Pape H. (2010). Alcohol, suppressed anger and violence. *Addiction* 105 1580–1586. 10.1111/j.1360-0443.2010.02997.x 20569229

[B27] Petersen S. E. Sporns O. (2015). Brain Networks and Cognitive Architectures. *Neuron* 88 207–219. 10.1016/j.neuron.2015.09.027 26447582 PMC4598639

[B28] Puddephatt J. A. Irizar P. Jones A. Gage S. H. Goodwin L. (2021). Associations of common mental disorder with alcohol use in the adult general population: A systematic review and meta-analysis. *Addiction* 117 1543–1572. 10.1111/add.15735 34729837 PMC9300028

[B29] Rubinov M. Sporns O. (2010). Complex network measures of brain connectivity: Uses and interpretations. *Neuroimage* 52 1059–1069. 10.1016/j.neuroimage.2009.10.003 19819337

[B30] Shine J. M. Poldrack R. A. (2018). Principles of dynamic network reconfiguration across diverse brain states. *Neuroimage* 180 396–405. 10.1016/j.neuroimage.2017.08.010 28782684

[B31] Sjoerds Z. Stufflebeam S. M. Veltman D. J. Van Den Brink W. Penninx B. W. Douw L. (2017). Loss of brain graph network efficiency in alcohol dependence. *Addict. Biol.* 22 523–534. 10.1111/adb.12346 26692359 PMC4917471

[B32] Smith L. C. Kimbrough A. (2020). Leveraging Neural Networks in Preclinical Alcohol Research. *Brain Sci.* 10:578. 10.3390/brainsci10090578 32825739 PMC7565429

[B33] Sonuga-Barke E. J. Castellanos F. X. (2007). Spontaneous attentional fluctuations in impaired states and pathological conditions: A neurobiological hypothesis. *Neurosci. Biobehav. Rev.* 31 977–986. 10.1016/j.neubiorev.2007.02.005 17445893

[B34] Spagnolli F. Cerini R. Cardobi N. Barillari M. Manganotti P. Storti S. (2013). Brain modifications after acute alcohol consumption analyzed by resting state fMRI. *Magn. Reson. Imaging* 31 1325–1330. 10.1016/j.mri.2013.04.007 23680187

[B35] Sporns O. Tononi G. KöTTER R. (2005). The human connectome: A structural description of the human brain. *PLoS Comput. Biol.* 1:e42. 10.1371/journal.pcbi.0010042 16201007 PMC1239902

[B36] Tononi G. Edelman G. M. Sporns O. (1998). Complexity and coherency: Integrating information in the brain. *Trends Cogn. Sci.* 2 474–484. 10.1016/s1364-6613(98)01259-5 21227298

[B37] Wang J. Wang X. Xia M. Liao X. Evans A. He Y. (2015). GRETNA: A graph theoretical network analysis toolbox for imaging connectomics. *Front. Hum. Neurosci.* 9:386. 10.3389/fnhum.2015.00386 26175682 PMC4485071

[B38] Watts D. J. Strogatz S. H. (1998). Collective dynamics of ‘small-world’ networks. *Nature* 393 440–442. 10.1038/30918 9623998

[B39] Yang H. Chen X. Chen Z. B. Li L. Li X. Y. Castellanos F. X. (2021). Disrupted intrinsic functional brain topology in patients with major depressive disorder. *Mol. Psychiatry* 26 7363–7371. 10.1038/s41380-021-01247-2 34385597 PMC8873016

[B40] Yoshida A. (1983). Differences in the isozymes involved in alcohol metabolism between caucasians and orientals. *Isozymes Curr. Top Biol. Med. Res.* 8 245–261. 6354999

[B41] Zheng H. Kong L. Chen L. Zhang H. Zheng W. (2015). Acute effects of alcohol on the human brain: A resting-state FMRI study. *Biomed. Res. Int.* 2015:947529. 10.1155/2015/947529 25705701 PMC4332461

