# Supplementary material for: Analysis on topological alterations of functional brain networks after acute alcohol intake using resting-state functional magnetic resonance imaging and graph theory
Source: Front Hum Neurosci. 2022 Sep 26;16:985986. doi: 10.3389/fnhum.2022.985986 (PMC9549745; doi:10.3389/fnhum.2022.985986)
Supplement: Supplementary file 1 [file Data_Sheet_1.pdf]

frontiers

Supplementary Material

Table S1 Regions of altered nodal clustering coefficient and nodal local efficiency in 1h Group and B Group.

Parameters	Nodle	Subnetwork	MNI X	MNI Y	MNI Z	p-value	t-value
1h Group < Control Group							
nodal clustering coefficient	sup frontal.R	Default	23	33	47	0.0005	-3.8989
	TPJ.L	Cingulo-opercular	-52	-63	15	0.0002	-4.1615
B Group < Control Group							
nodal clustering coefficient	ACC.R	Default	9	39	20	0.0024	-3.2958
	aPFC.L	Fronto-parietal	-29	57	10	0.0014	-3.4991
	dlPFC.R	Fronto-parietal	40	36	29	0.0001	-4.3322
	aPFC.R	Cingulo-opercular	27	49	26	0.0025	-3.2772
	dACC.R	Cingulo-opercular	9	20	34	0.0001	-4.3697
	vFC.L	Cingulo-opercular	-48	6	1	0.0019	-3.3929
	mid insula.R	Cingulo-opercular	37	-2	-3	0.0028	-3.2353
	TPJ.L	Cingulo-opercular	-52	-63	15	0.0023	-3.3118
	pre-SMA.R	Sensorimotor	10	5	51	0.0008	-3.7146
	frontal.R	Sensorimotor	53	-3	32	0.0005	-3.8783
	precentral gyrus.R	Sensorimotor	58	-3	17	0.0001	-4.5655
	precentral gyrus.R	Sensorimotor	46	-8	24	0.0002	-4.2032
	precentral gyrus.L	Sensorimotor	-54	-9	23	0.0004	-3.9274
	mid insula.R	Sensorimotor	33	-12	16	0.0003	-4.0018
	temporal.R	Sensorimotor	59	-13	8	0.0018	-3.4137
	med cerebellum.R	Cerebellum	14	-75	-21	0.0008	-3.6917
nodal local efficiency	dACC.R	Cingulo-opercular	9	20	34	0.0006	-3.8234
	frontal.R	Sensorimotor	53	-3	32	0.0004	-3.9324

	precentral gyrus.R	Sensorimotor	58	-3	17	0.0000	-5.1531
B Group < A Group							
nodal clustering coefficient	mid insula.R	Sensorimotor	33	-12	16	0.0008	-3.7149
	parietal.L	Sensorimotor	-38	-15	59	0.0003	-4.0316
	parietal.L	Sensorimotor	-24	-30	64	0.0005	-3.8398
	post occipital.R	Occipital	27	-91	2	0.0007	-3.7418
	lat cerebellum.L	Cerebellum	-28	-44	-25	0.0000	-4.8371
	lat cerebellum.L	Cerebellum	-24	-54	-21	0.0003	-4.0640
	med cerebellum.R	Cerebellum	5	-75	-11	0.0001	-4.3789

Comparisons between groups, nodal clustering coefficient and nodal local efficiency of some regions were decreased in 1h Group and B Group. Abbreviations: sup frontal, superior frontal gyrus; TPJ, temporo-parietal junction area; ACC, anterior cingulate cortex; aPFC, anterior prefrontal cortex; dACC, dorsal anterior cingulate cortex; vFC, ventral frontal cortex; mid insula, mid-insula; pre-SMA, presupplementary motor area; precentral, precentral gyrus; temporal, temporal lobe; med cerebellum, median cerebellum; parietal, parietal gyrus; post occipital, posterior occipital gyrus; lat cerebellum, lateral cerebellum.

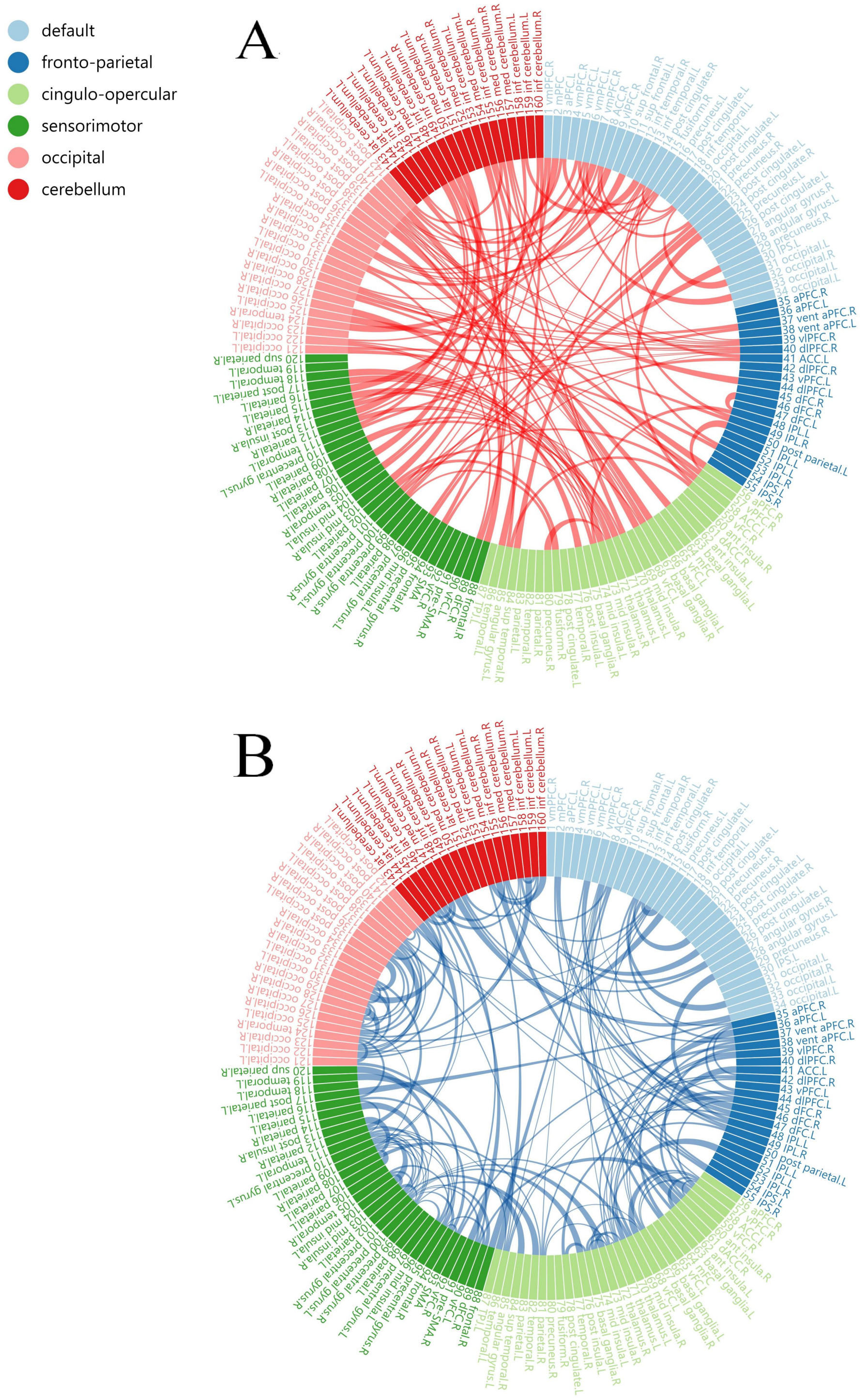

Figure S1. Group B showed two statistically different neural circuits compared to group A ($p < 0.05$, corrected). Edge colors represent increased (red) and decreased (blue) functional conneetivity strength in the volunteers. Edge sizes represent the level of significance. For more information about the functional connectivity changes, please refer to Supplementary File 3.
