# Supplementary material for: Analysis on topological alterations of functional brain networks after acute alcohol intake using resting-state functional magnetic resonance imaging and graph theory
Source: Front Hum Neurosci. 2022 Sep 26;16:985986. doi: 10.3389/fnhum.2022.985986 (PMC9549745; doi:10.3389/fnhum.2022.985986)
Supplement: Supplementary file 2 [file Table_1.DOCX]

Supplementary Material

**Information of ROI:** (Dosenbach Nico U F,Nardos Binyam,Cohen Alexander L,et al.Prediction of individual brain maturity using fMRI.SCIENCE.2010;329 (5997)：1358-61.)

In order to gain at least an estimation of the underlying functional area architecture, Dosenbach et al. chose to functionally define regions of interest from several meta-analyses of fMRI activation studies. A series of five meta-analyses, focused on error-processing, default-mode (task-induced deactivations), memory, language and sensorimotor functions were carried out.

For the sensorimotor and language meta-analyses, main effect of activation (ANOVA: time as single factor over 7 levels) statistical images, were generated from each included study. To identify voxels that were reliably activated across these studies, the activation images were thresholded at a high Z-score (Z > 7). The error (2-factor ANOVA: correctness (2-levels) x time (7-levels)) and memory-related (2-factor ANOVA: oldness (2-levels) x time (7-levels)) metaanalyses relied on statistically weaker interaction effects therefore the thresholds were scaled appropriately lower (error: Z > 4; memory: Z > 2) (N. U. F. Dosenbach et al., A core system for the implementation of task sets. Neuron 50, 799 (2006)). The default-mode regions were derived from meta-analyses of both trial-related (Z > 7; main effect of deactivation) and sustained deactivations (Z > 2). As for the analysis of interactions a lower threshold was applied to the sustained signals since they are statistically less reliable. Several thresholds were tested initially, and the peaks identified were very similar. Additionally, there was substantial overlap in identified peaks between meta-analyses, and so small differences in peaks identified from a single meta-analysis were offset by redundancies with other meta-analyses.

Voxels passing the threshold were set equal to one and all others equal to zero, creating a mask of significantly activated voxels for each study. The masks for all studies were summed to create a conjunction image, where each voxel carried a value between zero and the number of studies in the meta-analysis, indicating how often voxels were significantly activated across the meta-analysis. After applying a 4 mm smoothing kernel to these images, peak-finding algorithms were used to identify centroids of reliably activated groups of voxels.

Ten mm diameter spheres were centered at the activity peaks for each meta-analysis. ROIs from the separate meta-analyses were then combined by giving priority to ROIs identified by task-induced deactivations (default-mode) and error > correct (control). For overlapping ROIs across the language, memory and sensorimotor meta-analyses, a spatial average location was first computed. Language, memory and sensorimotor-related ROIs were then added at the final step. Previously published cognitive control regions (N. U. F. Dosenbach et al., Distinct brain networks for adaptive and stable task control in humans. Proc. Natl. Acad. Sci. U.S.A. 104, 11073 (2007); N. U. F. Dosenbach, D. A. Fair, A. L. Cohen, B. L. Schlaggar, S. E. Petersen, A dualnetworks architecture of top-down control. Trends Cogn. Sci. 12, 99 (2008)) were included in the set of errorprocessing related regions.

In this manner, a total of 160 ROIs were generated. The distance between all ROI centers was at least 10 mm, thus not allowing any spatial overlap between ROIs. All three-dimensional coordinates reported in the manuscript are in MNI space.

**Information of** **Dosenbach 160**

|  |  |  |  | **MNI-coordinates** | | |
| --- | --- | --- | --- | --- | --- | --- |
| **Labels** | **Regions** | **Hemishpere** | **Subnetwork** | **x(mm)** | **y(mm)** | **z(mm)** |
| 1 | vmPFC | R | default | 6 | 64 | 3 |
| 2 | mPFC |  | default | 0 | 51 | 32 |
| 3 | aPFC | L | default | -25 | 51 | 27 |
| 4 | vmPFC | R | default | 9 | 51 | 16 |
| 5 | vmPFC | L | default | -6 | 50 | -1 |
| 6 | vmPFC | L | default | -11 | 45 | 17 |
| 7 | vmPFC | R | default | 8 | 42 | -5 |
| 8 | ACC | R | default | 9 | 39 | 20 |
| 9 | vlPFC | R | default | 46 | 39 | -15 |
| 10 | sup frontal | R | default | 23 | 33 | 47 |
| 11 | sup frontal | L | default | -16 | 29 | 54 |
| 12 | inf temporal | R | default | 52 | -15 | -13 |
| 13 | inf temporal | L | default | -59 | -25 | -15 |
| 14 | post cingulate | R | default | 1 | -26 | 31 |
| 15 | fusiform | R | default | 28 | -37 | -15 |
| 16 | precuneus | L | default | -3 | -38 | 45 |
| 17 | post cingulate | L | default | -8 | -41 | 3 |
| 18 | inf temporal | L | default | -61 | -41 | -2 |
| 19 | occipital | L | default | -28 | -42 | -11 |
| 20 | post cingulate | L | default | -5 | -43 | 25 |
| 21 | precuneus | R | default | 9 | -43 | 25 |
| 22 | precuneus | R | default | 5 | -50 | 33 |
| 23 | post cingulate | L | default | -5 | -52 | 17 |
| 24 | post cingulate | R | default | 10 | -55 | 17 |
| 25 | precuneus | L | default | -6 | -56 | 29 |
| 26 | post cingulate | L | default | -11 | -58 | 17 |
| 27 | angular gyrus | R | default | 51 | -59 | 34 |
| 28 | angular gyrus | L | default | -48 | -63 | 35 |
| 29 | precuneus | R | default | 11 | -68 | 42 |
| 30 | IPS | L | default | -36 | -69 | 40 |
| 31 | occipital | L | default | -9 | -72 | 41 |
| 32 | occipital | R | default | 45 | -72 | 29 |
| 33 | occipital | L | default | -2 | -75 | 32 |
| 34 | occipital | L | default | -42 | -76 | 26 |
| 35 | aPFC | R | fronto-parietal | 29 | 57 | 18 |
| 36 | aPFC | L | fronto-parietal | -29 | 57 | 10 |
| 37 | vent aPFC | R | fronto-parietal | 42 | 48 | -3 |
| 38 | vent aPFC | L | fronto-parietal | -43 | 47 | 2 |
| 39 | vlPFC | R | fronto-parietal | 39 | 42 | 16 |
| 40 | dlPFC | R | fronto-parietal | 40 | 36 | 29 |
| 41 | ACC | L | fronto-parietal | -1 | 28 | 40 |
| 42 | dlPFC | R | fronto-parietal | 46 | 28 | 31 |
| 43 | vPFC | L | fronto-parietal | -52 | 28 | 17 |
| 44 | dlPFC | L | fronto-parietal | -44 | 27 | 33 |
| 45 | dFC | R | fronto-parietal | 40 | 17 | 40 |
| 46 | dFC | R | fronto-parietal | 44 | 8 | 34 |
| 47 | dFC | L | fronto-parietal | -42 | 7 | 36 |
| 48 | IPL | L | fronto-parietal | -41 | -40 | 42 |
| 49 | IPL | R | fronto-parietal | 54 | -44 | 43 |
| 50 | post parietal | L | fronto-parietal | -35 | -46 | 48 |
| 51 | IPL | L | fronto-parietal | -48 | -47 | 49 |
| 52 | IPL | L | fronto-parietal | -53 | -50 | 39 |
| 53 | IPL | R | fronto-parietal | 44 | -52 | 47 |
| 54 | IPS | L | fronto-parietal | -32 | -58 | 46 |
| 55 | IPS | R | fronto-parietal | 32 | -59 | 41 |
| 56 | aPFC | R | cingulo-opercular | 27 | 49 | 26 |
| 57 | vPFC | R | cingulo-opercular | 34 | 32 | 7 |
| 58 | ACC | L | cingulo-opercular | -2 | 30 | 27 |
| 59 | vFC | R | cingulo-opercular | 51 | 23 | 8 |
| 60 | ant insula | R | cingulo-opercular | 38 | 21 | -1 |
| 61 | dACC | R | cingulo-opercular | 9 | 20 | 34 |
| 62 | ant insula | L | cingulo-opercular | -36 | 18 | 2 |
| 63 | basal ganglia | L | cingulo-opercular | -6 | 17 | 34 |
| 64 | mFC |  | cingulo-opercular | 0 | 15 | 45 |
| 65 | vFC | L | cingulo-opercular | -46 | 10 | 14 |
| 66 | basal ganglia | L | cingulo-opercular | -20 | 6 | 7 |
| 67 | basal ganglia | R | cingulo-opercular | 14 | 6 | 7 |
| 68 | vFC | L | cingulo-opercular | -48 | 6 | 1 |
| 69 | mid insula | R | cingulo-opercular | 37 | -2 | -3 |
| 70 | thalamus | L | cingulo-opercular | -12 | -3 | 13 |
| 71 | thalamus | L | cingulo-opercular | -12 | -12 | 6 |
| 72 | thalamus | R | cingulo-opercular | 11 | -12 | 6 |
| 73 | mid insula | R | cingulo-opercular | 32 | -12 | 2 |
| 74 | mid insula | L | cingulo-opercular | -30 | -14 | 1 |
| 75 | basal ganglia | R | cingulo-opercular | 11 | -24 | 2 |
| 76 | post insula | L | cingulo-opercular | -30 | -28 | 9 |
| 77 | temporal | R | cingulo-opercular | 51 | -30 | 5 |
| 78 | post cingulate | L | cingulo-opercular | -4 | -31 | -4 |
| 79 | fusiform | R | cingulo-opercular | 54 | -31 | -18 |
| 80 | precuneus | R | cingulo-opercular | 8 | -40 | 50 |
| 81 | parietal | R | cingulo-opercular | 58 | -41 | 20 |
| 82 | temporal | R | cingulo-opercular | 43 | -43 | 8 |
| 83 | parietal | L | cingulo-opercular | -55 | -44 | 30 |
| 84 | sup temporal | R | cingulo-opercular | 42 | -46 | 21 |
| 85 | angular gyrus | L | cingulo-opercular | -41 | -47 | 29 |
| 86 | temporal | L | cingulo-opercular | -59 | -47 | 11 |
| 87 | TPJ | L | cingulo-opercular | -52 | -63 | 15 |
| 88 | frontal | R | sensorimotor | 58 | 11 | 14 |
| 89 | dFC | R | sensorimotor | 60 | 8 | 34 |
| 90 | vFC | L | sensorimotor | -55 | 7 | 23 |
| 91 | pre-SMA | R | sensorimotor | 10 | 5 | 51 |
| 92 | vFC | R | sensorimotor | 43 | 1 | 12 |
| 93 | SMA |  | sensorimotor | 0 | -1 | 52 |
| 94 | frontal | R | sensorimotor | 53 | -3 | 32 |
| 95 | precentral gyrus | R | sensorimotor | 58 | -3 | 17 |
| 96 | mid insula | L | sensorimotor | -42 | -3 | 11 |
| 97 | precentral gyrus | L | sensorimotor | -44 | -6 | 49 |
| 98 | parietal | L | sensorimotor | -26 | -8 | 54 |
| 99 | precentral gyrus | R | sensorimotor | 46 | -8 | 24 |
| 100 | precentral gyrus | L | sensorimotor | -54 | -9 | 23 |
| 101 | precentral gyrus | R | sensorimotor | 44 | -11 | 38 |
| 102 | parietal | L | sensorimotor | -47 | -12 | 36 |
| 103 | mid insula | R | sensorimotor | 33 | -12 | 16 |
| 104 | mid insula | L | sensorimotor | -36 | -12 | 15 |
| 105 | temporal | R | sensorimotor | 59 | -13 | 8 |
| 106 | parietal | L | sensorimotor | -38 | -15 | 59 |
| 107 | parietal | L | sensorimotor | -47 | -18 | 50 |
| 108 | parietal | R | sensorimotor | 46 | -20 | 45 |
| 109 | parietal | L | sensorimotor | -55 | -22 | 38 |
| 110 | precentral gyrus | L | sensorimotor | -54 | -22 | 22 |
| 111 | temporal | L | sensorimotor | -54 | -22 | 9 |
| 112 | parietal | R | sensorimotor | 41 | -23 | 55 |
| 113 | post insula | R | sensorimotor | 42 | -24 | 17 |
| 114 | parietal | R | sensorimotor | 18 | -27 | 62 |
| 115 | parietal | L | sensorimotor | -38 | -27 | 60 |
| 116 | parietal | L | sensorimotor | -24 | -30 | 64 |
| 117 | post parietal | L | sensorimotor | -41 | -31 | 48 |
| 118 | temporal | L | sensorimotor | -41 | -37 | 16 |
| 119 | temporal | L | sensorimotor | -53 | -37 | 13 |
| 120 | sup parietal | R | sensorimotor | 34 | -39 | 65 |
| 121 | occipital | L | occipital | -18 | -50 | 1 |
| 122 | occipital | L | occipital | -34 | -60 | -5 |
| 123 | occipital | R | occipital | 36 | -60 | -8 |
| 124 | temporal | R | occipital | 46 | -62 | 5 |
| 125 | occipital | L | occipital | -44 | -63 | -7 |
| 126 | occipital | R | occipital | 19 | -66 | -1 |
| 127 | occipital | R | occipital | 17 | -68 | 20 |
| 128 | occipital | R | occipital | 39 | -71 | 13 |
| 129 | occipital | R | occipital | 29 | -73 | 29 |
| 130 | occipital | L | occipital | -29 | -75 | 28 |
| 131 | occipital | L | occipital | -16 | -76 | 33 |
| 132 | occipital | R | occipital | 9 | -76 | 14 |
| 133 | occipital | R | occipital | 15 | -77 | 32 |
| 134 | occipital | R | occipital | 20 | -78 | -2 |
| 135 | post occipital | L | occipital | -5 | -80 | 9 |
| 136 | post occipital | R | occipital | 29 | -81 | 14 |
| 137 | post occipital | R | occipital | 33 | -81 | -2 |
| 138 | post occipital | L | occipital | -37 | -83 | -2 |
| 139 | post occipital | L | occipital | -29 | -88 | 8 |
| 140 | post occipital | R | occipital | 13 | -91 | 2 |
| 141 | post occipital | R | occipital | 27 | -91 | 2 |
| 142 | post occipital | L | occipital | -4 | -94 | 12 |
| 143 | lat cerebellum | L | cerebellum | -28 | -44 | -25 |
| 144 | lat cerebellum | L | cerebellum | -24 | -54 | -21 |
| 145 | inf cerebellum | L | cerebellum | -37 | -54 | -37 |
| 146 | lat cerebellum | L | cerebellum | -34 | -57 | -24 |
| 147 | med cerebellum | L | cerebellum | -6 | -60 | -15 |
| 148 | inf cerebellum | L | cerebellum | -25 | -60 | -34 |
| 149 | inf cerebellum | R | cerebellum | 32 | -61 | -31 |
| 150 | med cerebellum | L | cerebellum | -16 | -64 | -21 |
| 151 | lat cerebellum | R | cerebellum | 21 | -64 | -22 |
| 152 | med cerebellum | R | cerebellum | 1 | -66 | -24 |
| 153 | inf cerebellum | L | cerebellum | -34 | -67 | -29 |
| 154 | med cerebellum | L | cerebellum | -11 | -72 | -14 |
| 155 | inf cerebellum | R | cerebellum | 33 | -73 | -30 |
| 156 | med cerebellum | R | cerebellum | 5 | -75 | -11 |
| 157 | med cerebellum | R | cerebellum | 14 | -75 | -21 |
| 158 | inf cerebellum | L | cerebellum | -21 | -79 | -33 |
| 159 | inf cerebellum | L | cerebellum | -6 | -79 | -33 |
| 160 | inf cerebellum | R | cerebellum | 18 | -81 | -33 |
|  |  |  |  |  |  |  |
| **This set of ROIs are from Dosenbach et al., 2010, Science (Sphere, r = 5mm)** | | | |  |  |  |
|  |  |  |  |  |  |  |
